# Comprehensive molecular-genetic analysis of mid-frequency sensorineural hearing loss

**DOI:** 10.1038/s41598-021-01876-1

**Published:** 2021-11-18

**Authors:** Zuzana Pavlenkova, Lukas Varga, Silvia Borecka, Miloslav Karhanek, Miloslava Huckova, Martina Skopkova, Milan Profant, Daniela Gasperikova

**Affiliations:** 1 Department of Otorhinolaryngology-Head and Neck Surgery, Faculty of Medicine and University Hospital, Comenius University, Bratislava, Slovakia; 2 DIABGENE Laboratory, Biomedical Research Center, Slovak Academy of Sciences, Bratislava, Slovakia; 3 Laboratory of Bioinformatics, Biomedical Research Center, Slovak Academy of Sciences, Bratislava, Slovakia

**Keywords:** Clinical genetics, Mutation, Clinical genetics, Genetic testing, Alport syndrome, Genetics, Diseases, Genetics research

## Abstract

The genetic heterogeneity of sensorineural hearing loss (SNHL) is a major hurdle to the detection of disease-causing variants. We aimed to identify underlying causal genes associated with mid-frequency hearing loss (HL), which contributes to less than about 1% of SNHL cases, by whole exome sequencing (WES). Thirty families segregating mid-frequency SNHL, in whom biallelic *GJB2* mutations had been previously excluded, were selected from among 851 families in our DNA repository of SNHL. DNA samples from the probands were subjected to WES analysis and searched for candidate variants associated with SNHL. We were able to identify the genetic aetiology in six probands (20%). In total, we found three pathogenic and three likely pathogenic variants in four genes (*COL4A5*, *OTOGL*, *TECTA*, *TMPRSS3*). One more proband was a compound heterozygote for a pathogenic variant and a variant of uncertain significance (VUS) in *MYO15A* gene. To date, *MYO15A* and *TMPRSS3* have not yet been described in association with mid-frequency SNHL. In eight additional probands, eight candidate VUS variants were detected in five genes (*DIAPH1*, *MYO7A*, *TECTA*, *TMC1*, *TSPEAR*). Seven of these 16 variants have not yet been published or mentioned in the available databases. The most prevalent gene was *TECTA*, identified in 23% of all tested families. Furthermore, we confirmed the hypothesis that a substantive portion of cases with this conspicuous audiogram shape is a consequence of a genetic disorder.

## Introduction

Hearing loss (HL) is the most common sensory deficit, affecting 466 million people (5% of the world’s population) and with an incidence of 1.45/1000 newborns^1,2^. At least half of congenital deafness cases is due to genetic factors, the vast majority of which is monogenic. In syndromic HL, a specific phenotype may narrow the selection of candidate genes for targeted genetic analysis. However, in the case of non-syndromic hearing impairment, such preselection of candidate genes is very limited. Non-syndromic hereditary HL is also characterized by extreme genetic heterogeneity. More than 6000 mutations in more than 150 genes have been causally implicated in deafness^3^. This underscores the importance of the use of massively parallel sequencing for genetic diagnostics of HL.

Pure tone audiometry (0.25–8 kHz) is the gold standard for measuring hearing in older children and adults^4^. It allows determination of the degree of hearing impairment, assigns it to a specific type of audiometric curve and enables characterization of the distinct shapes of this curve (basocochlear, mediocochlear, pancochlear, apicocochlear, ski slope, etc.). The shape of the audiometric curve may also be indicative of certain diseases which commonly manifest by HL, e.g. the Carhart notch in otosclerosis^5^, the low-frequency HL found initially in Ménière's disease^6^ or in children with cytomegalovirus infection^7,8^.

Based on a detailed analysis of audiograms and the patient’s age, it is sometimes possible to predict the probable genetic cause of hearing impairment in patients with non-syndromic HL^9^. The high-frequency (basocochlear, descending) and pancochlear (flat) types of the audiometric curve dominate in clinical practice. On the other hand, audiograms characteristic for mid-frequency (mediocochlear, cookie-bite or U-shaped) and low-frequency (apicocochlear or ascending) hearing impairment are rare. Mid-frequency HL accounts for only about 0.7–1% of all SNHL cases^10–12^, and the majority of this HL is believed to have genetic aetiology^13^.

The aim of our study was to identify causative gene variants associated with mid-frequency hearing loss.

## Results

Using WES, we analyzed 30 families with mid-frequency HL, which represented 4.95% of all *GJB2* unrelated SNHL cases in our cohort. We established the genetic aetiology of HL in six families (20%) and we assumed the cause of HL based on the detected candidate VUS variants in an additional eight families (27%). A compound heterozygote state of a pathogenic and a VUS variant was observed in one family (Table 1). Sixteen variants were found in total, seven of which are novel, i.e. they are not yet present in available deafness databases or the published literature. All detected variants occurred in genes known to be associated with HL (Table 2).

**Table 1:** Phenotype in 15 probands with mid-frequency SNHL.

Family	Proband	Family history	Proband's phenotype	Degree of HL and pure tone average	Age at onset	Progression of HL	Gene
FAM618	D815	Familial	Microscopic haematuria chronic glomerulonephritis	Moderate 46 dB	Postlingual	Progressive	*COL4A5*
FAM806	D1248	Familial	Non-syndromic HL	Moderately-severe 57 dB	4,5 years	Progressive	*DIAPH1*
FAM411	D520	Familial	Non-syndromic HL	Mild R: 25 dB; L: 38 dB	22 years	Mild progressive	*MYO7A*
FAM837	D1321	Sporadic	Non-syndromic HL	Profound 109 dB	1,5 year	Progressive	*MYO15A*
FAM834	D1315	Sporadic	Non-syndromic HL	Moderate 48 dB	Congenital	Stable	*OTOGL*
FAM417	D528	N/A	Non-syndromic HL	Mild 40 dB	16 years	Progressive	*TECTA*
FAM642	D847	Familial	Non-syndromic HL	Moderate 49 dB	Postlingual	Progressive	*TECTA*
FAM622	D821	Familial	Non-syndromic HL	Severe 81 dB	N/A	Progressive	*TECTA*
FAM571	D753	Familial	Non-syndromic HL	Moderate 41 dB	11 years	Progressive	*TECTA*
FAM623	D822	Familial	Non-syndromic HL	Moderate 53 dB	5 years	Mild progressive	*TECTA*
FAM090	D103	N/A	Non-syndromic HL	Mild R: 26 dB; L: 31 dB	N/A	N/A	*TECTA*
FAM200	D231	N/A	Non-syndromic HL	R: moderate 51 dB; L: moderately-severe 66 dB	Congenital	Mild progressive	*TECTA*
FAM416	D527	Familial	Non-syndromic HL	Mild 29 dB	30 years	Progressive	*TMC1*
FAM253	D298	Sporadic	Non-syndromic HL	Moderately-severe 69 dB	Perilingual	Progressive	*TMPRSS3*
FAM174	D197	Familial	N/A	Severe 81 dB	N/A	N/A	*TSPEAR*

N/A, not available.

**Table 2:** Pathogenicity evaluation of 16 variants based on in-depth ACMG HL guideline application in 15 probands with mid-frequency SNHL.

Family	Proband	Family history	Gene	Genomic position GRCh38	Transcript	Exon	c.DNA position	Protein position	Zygosity	Global frequency	Max frequency	Popmax AF population	CADD	REVEL	ACMG class	rs number	Reference
FAM618	D815	familial	*COL4A5*	ChrX:g.108591636G>A	NM_000495.5	21/51	c.1415G>A	p.Gly472Glu	1	0	0	0	24.1	0.9559	Likely pathogenic PM1, PM2, PM5, PP1, PP3, PP4	N/A	This study
FAM806	D1248	familial	*DIAPH1*	Chr5:g.141524214C>T	NM_005219.5	27/28	c.3590G>A	p.Gly1197Asp	0/1	0	0	0	31.0	0.841	VUS PM2, PP1, PP3	rs1354335913	This study*
FAM411	D520	familial	*MYO7A*	Chr11:g.77192139G>A	NM_000260.4	31/49	c.4013G>A	p.Arg1338His	0/1	0.0000197	0.00006539 Latino/Admixed American	0.000004880 European (non-Finnish)	30.0	0.7559	VUS PM2, PP3	rs778477059	This study*
FAM837	D1321	sporadic	*MYO15A*	Chr17:g.18142122G>A	NM_016239.4	24/66	c.5693G>A	p.Arg1898Gln	0/1	0.00000657	0.00001470 European (non-Finnish)	0	27.6	0.901	VUS PM2, PM3, PP3	rs756752580	This study*
			*MYO15A*	Chr17:g.18153858T>C	NM_016239.4	43/66	c.8050 T>C	p.Tyr2684His	0/1	0.00006572	0.001729 Ashkenazi Jewish	0.00001171 European (non-Finnish)	26.2	0.837	Pathogenic PM3_S, PP1_S, PP3, BS1_P	rs376351191	^57^*
FAM834	D1315	sporadic	*OTOGL*	Chr12:g.80255183C>T	NM_173591.5	16/59	c.1558C>T	p.Gln520*	0/1	0.00006578	0.0001178 European (non-Finnish)	0.00005850 European (non-Finnish)	49.0	N/A	Pathogenic PVS1, PM3, PM2_P	rs371465450	^42^*
			*OTOGL*	Chr12:g.80320678(53 bp)del	NM_173591.5	34/59 (intron 34/58)	c.4032_4054 + 30del	p.?	0/1	0	0	0	N/A	N/A	Likely pathogenic PVS1_S, PM2, PM3_P	N/A	This study
FAM417	D528	N/A	*TECTA*	Chr11:g.121127925 T>C	NM_005422.4	9/24	c.1948 T>C	p.Cys650Arg	0/1	0	0	0	28.1	0.975	VUS PM2, PP3	N/A	This study
FAM642	D847	familial	*TECTA*	Chr11:g.121166767G>A	NM_005422.4	18/24	c.5573G>A	p.Gly1858Glu	0/1	0	0	0	32.0	0.8169	VUS PM2, PP3	N/A	This study
FAM622	D821	familial	*TECTA*	Chr11:g.121168064C>T	NM_005422.4	19/24	c.5597C>T	p.Thr1866Met	0/1	0.000006572	0.00001470 European (non-Finnish)	0	30.0	0.7609	Pathogenic PS4, PP1_S, PM2, PP3	rs140236996	^91^*
FAM571	D753	familial	*TECTA*	Chr11:g.121168135C>T	NM_005422.4	19/24	c.5668C>T	p.Arg1890Cys	0/1	0	0	0	27.8	0.7139	Pathogenic PP1_S, PM2, PS2_M, PS4_M, PP3	rs121909063	^92^*
FAM623	D822	familial	*TECTA*	Chr11:g.121168135C>T	NM_005422.4	19/24	c.5668C>T	p.Arg1890Cys	0/1	0	0	0	27.8	0.7139	Pathogenic PP1_S, PM2, PS2_M, PS4_M, PP3	rs121909063	^92^*
FAM090	D103	N/A	*TECTA*	Chr11:g.121168751A>G	NM_005422.4	20/24	c.5825A>G	p.Tyr1942Cys	0/1	0	0	0	29.6	0.896	VUS PM2, PP3	rs373343675	This study
FAM200	D231	N/A	*TECTA*	Chr11:g.121190788insTA	NM_005422.4	24/24	c.6449_6450insTA	p.Lys2150Asnfs*9	0/1	0	0	0	N/A	N/A	VUS PM2, PM4	N/A	This study
FAM416	D527	familial	*TMC1*	Chr9:g.72821027C>T	NM_138691.3	20/24	c.1949C>T	p.Pro650Leu	0/1	0	0	0	26.9	0.54	VUS PM2	N/A	This study
FAM253	D298	sporadic	*TMPRSS3*	Chr21:g.42388935G>A	NM_024022.3	4/13	c.316C>T	p.Arg106Cys	1/1	0.0001051	0.0004146 South Asian	0.00009046 European (non-Finnish)	27.9	0.7419	Likely pathogenic PM3, PP1_M, PP3, PM2_P	rs139805921	^70^*
FAM174	D197	familial	*TSPEAR*	Chr21:g.44499923C>A	NM_144991.3	12/12	c.1870G>T	p.Glu624*	1/1	0.00001314	0.00002415 African/African-American	0	49.0	N/A	VUS PVS1_M, PM2_P, PM3_P	rs587717339	This study*

*, variant occurs in https://deafnessvariationdatabase.org/; _M, moderate; _P, supporting; _S, strong; N/A, not available; VUS, variants of uncertain significance.

### Dominant variants

**Figure 1:**
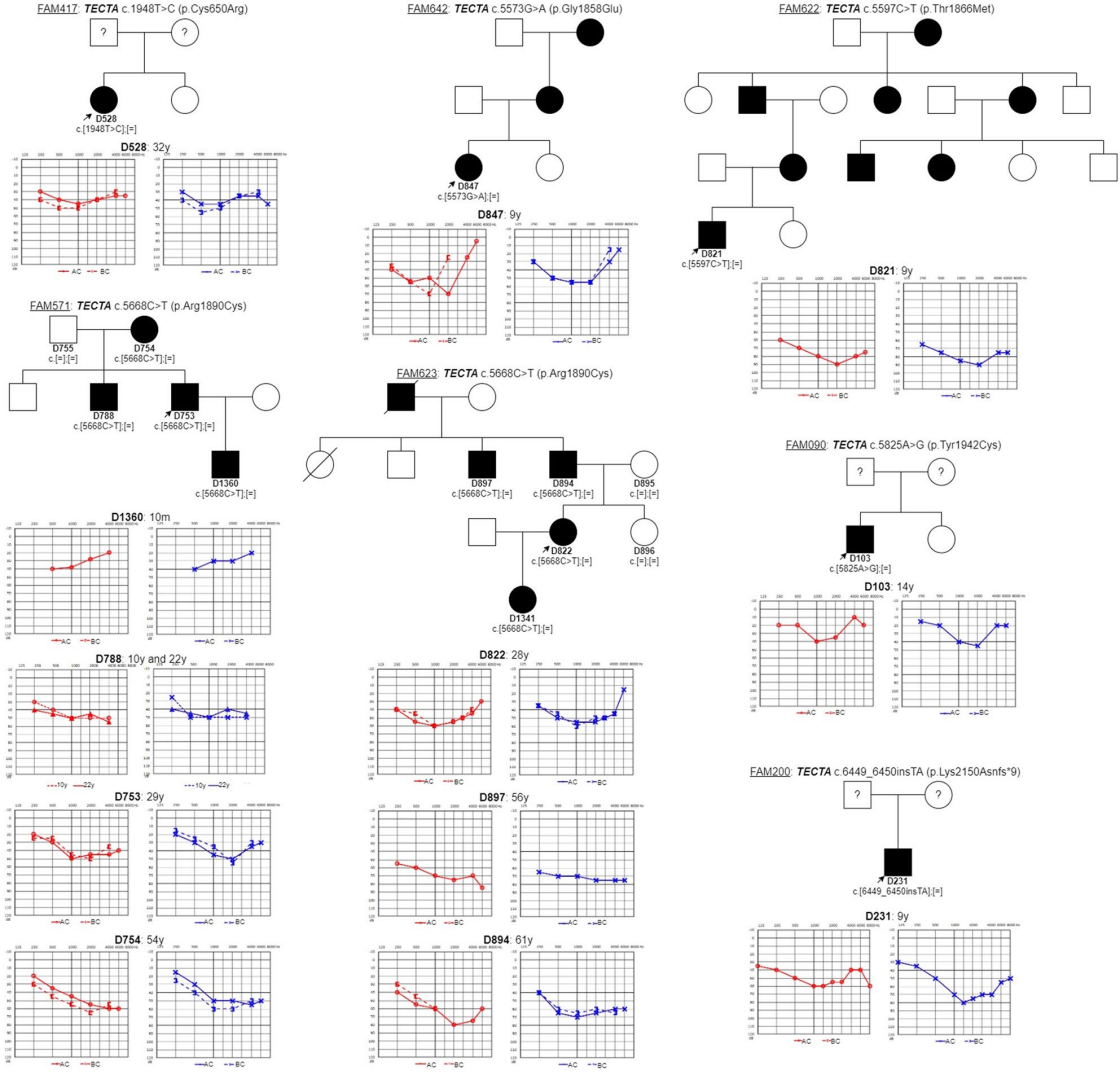
Pedigrees and audiograms of the patients with pathogenic and VUS variants in *TECTA* gene. The age at examination by pure tone audiometry is indicated. The youngest subject D1360 (born in 2018) from family FAM571 was only investigated by Auditory Steady-State Responses.

The most prevalent gene was *TECTA* (NM_005422.4) with five autosomal dominant missense variants (p.Cys650Arg, p.Gly1858Glu, p.Thr1866Met, p.Arg1890Cys, and p.Tyr1942Cys) and one frameshift variant (p.Lys2150Asnfs*9) detected in the heterozygous form in seven families. The pathogenic p.Arg1890Cys variant occurred in two families (FAM571, FAM623) (Fig. 1) and caused mild to moderately-severe HL. Over the years, the audiogram had a tendency to flatten in some of the affected family members. Another pathogenic variant, p.Thr1866Met, and the VUS p.Gly1858Glu variant were identified in families FAM622 and FAM642, respectively. Both families had several family members suffering from HL. They were not available for genetic testing, but the pedigrees suggest the presence of the autosomal dominant pattern of inheritance. In three families (FAM090, FAM200, and FAM417) only the proband’s DNA sample and reliable clinical data were available.

**Figure 2:**
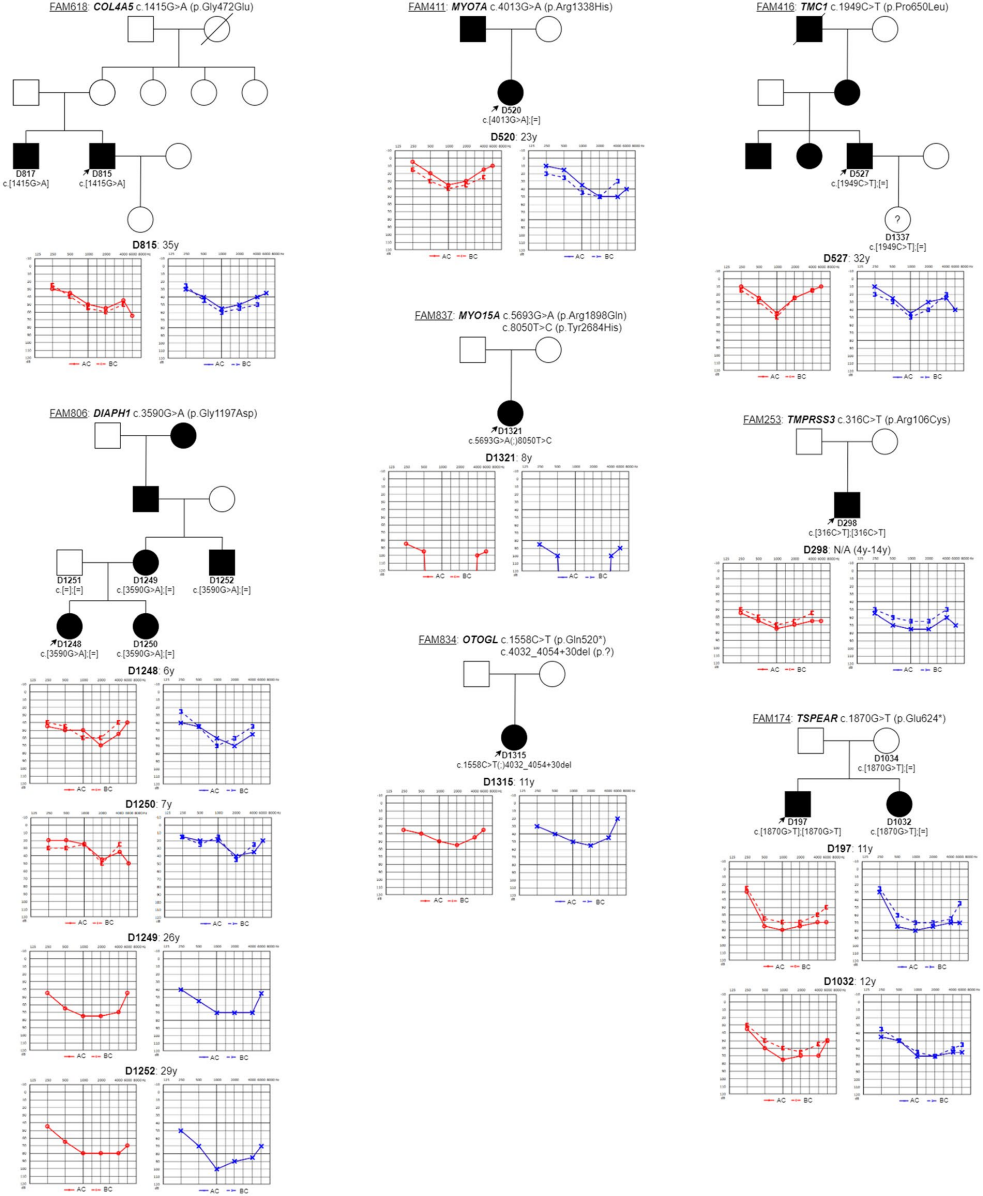
Pedigrees and audiograms of the patients with dominant variants in genes *DIAPH1*, *MYO7A*, *TMC1*, recessive variants in genes *MYO15A*, *OTOGL*, *TMPRSS3*, *TSPEAR* and variant with X-linked inheritance (*COL4A5*). The age at examination by pure tone audiometry is indicated.

Dominant variants (all belonging to the VUS category) in other genes were detected in three families (Fig. 2). In family FAM806 we identified a novel p.Gly1197Asp variant in the *DIAPH1* gene (NM_005219.5) in several family members. Hearing impairment in this family manifested in the age ranging from 2 to 15 years. A novel p.Pro650Leu variant in the *TMC1* (NM_138691.3) gene was identified in an adult patient who self-reported HL onset by the age of 30 years. His twelve-year-old daughter, who also carries the p.Pro650Leu variant, still has normal hearing but may develop HL in the future, similarly as the proband. The last dominant p.Arg1338His variant in the *MYO7A* gene (NM_000260.4) was found in family FAM411, with late HL onset.

### Recessive variants

We detected six recessive variants in genes *MYO15A*, *OTOGL*, *TMPRSS3* and *TSPEAR* in four families (27%) (Fig. 2). Three families (FAM253, FAM834 and FAM837) were characterized by sporadic HL. Familial HL occurred in FAM174, with two affected siblings. We identified a pathogenic and VUS variant in the *MYO15A* gene (NM_016239.4) in family FAM837, which was characterized with early-onset HL. Due to rapid HL progression, the proband received a cochlear implant at the age of 9 years with excellent early outcomes (a free field pure tone average 38.75 dB at 10 months post-implantation). One pathogenic variant and one likely pathogenic variant in the *OTOGL* gene (NM_173591.5) were detected in the proband D1315 (family FAM834), who had congenital HL. In both families, it would be appropriate to investigate the genotype of the parents to exclude the cis form of the variants in the probands.

The two remaining families carrying homozygous variants are of Roma ethnicity. In family FAM253, with perilingual HL, we detected the known p.Arg106Cys variant in the *TMPRSS3* gene (NM_024022.3). From a clinical point of view, the proband’s hearing gradually deteriorated to deafness between the 4th and 14th year of life. In the last family, FAM174, we detected VUS variant p.Glu624* in the *TSPEAR* gene (NM_144991.3). The proband’s sister (D1032) is also affected by HL but interestingly was found to only be a heterozygous carrier of the p.Glu624* variant. The HL phenotype was similar in both siblings.

### X-linked

In family FAM618 we identified a novel variant p.Gly472Glu in the *COL4A5* gene (NM_000495.5) in two affected siblings (D815, D817), both of whom showed a clinical picture of Alport syndrome. In addition to HL, they had both suffered from microscopic haematuria since childhood as well as symptoms of chronic glomerulonephritis. In subject D817, Alport syndrome was confirmed by kidney biopsy and the condition has progressively led to end-stage kidney failure with the need for haemodialysis.

## Discussion

Mutations in *GJB2* have been reported as the most frequent cause of hereditary HL^14,15^. For this reason, we first analyzed the *GJB2* gene by Sanger sequencing in all 851 probands. Using this method, we determined the genetic aetiology in 245 (29%) of the probands, who were then excluded from further analyses.

In the next step, the remaining probands were selected based on the clinical phenotype. Although we focused on a relatively uniform group of patients from the audiological point of view (mid-frequency HL), the number of potentially involved genes was large (> 226). Moreover, the spectrum of the genes responsible for this particular form of HL is mostly unknown. It is not practical to analyze each gene separately in such a case. Therefore, next generation sequencing (NGS) allowing the analysis of many genes simultaneously seemed to be the best solution^16,17^.

Using WES, we identified the genetic aetiology in six of the 30 probands (20%) with mid-frequency HL. Additionally, novel candidate variants (VUS) were found in eight probands. A compound heterozygous pathogenic and a VUS variant were observed in one proband. The detected variants were found in nine genes (*COL4A5*, *DIAPH1*, *MYO7A*, *MYO15A*, *OTOGL*, *TECTA*, *TMC1*, *TMPRSS3*, *TSPEAR*) harbouring four pathogenic variants, three likely pathogenic variants and nine VUS variants associated with hearing impairment. Seven of these variants have not yet been described in the literature or have not yet been submitted to deafness databases (Table 2). Five of the identified genes (*MYO7A*, *MYO15A*, *TMC1*, *TMPRSS3* and *TSPEAR*) have not yet been reported as a cause of mid-frequency SNHL. Other genes which are known to cause non-syndromic mid-frequency HL but were not detected in our cohort include: *CCDC50*^18^, *COL11A1*^19^, *COL11A2*^20,21^, *EYA4*^22^, *KCNQ4*^23^, *OTOA*^24^ and *POU4F3*^25^. Among these genes, we were able to identify three distinct groups based on the site of their expression and their function^18,26–29^.

### Glycoproteins expressed in the tectorial membrane

The tectorial membrane (TM) of the cochlea is a ribbon-like strip of extracellular matrix that contacts the tips of the sensory hair bundles of the outer hair cells. Sound induces movement of these hair cells relative to the TM, deflects the stereocilia, and leads to fluctuations in hair-cell membrane potential, transducing sound into electrical signals^30,31^.

The *TECTA* gene encodes a protein called alpha-tectorin. Mutations in *TECTA* account for 2–4% of all autosomal dominant non-syndromic SNHL characterized by mild prelingual HL from a clinical point of view^15,32–34^. TECTA contains several extracellular matrix (ECM)-interacting domains, including a nidogen-like domain and von Willebrand factor type D domains (vWFDs), which may allow binding to collagens and other glycoproteins, and a zona pellucida (ZP) domain important for the multimerization and stability of the protein. Missense mutations in different Tecta domains are known to cause different phenotypes with distinct changes in the structure of the tectorial membrane. Mutations in the zona pellucida domain cause mid-frequency HL with a typical U-shaped or shallow U-shaped audiogram, whereas mutations in the zonadhesin-like domain comprising vWFD1, vWFD2 and trypsin inhibitor-like (TIL) repeats lead to high-frequency HL^35,36,37^. Our results are in agreement with the findings of previous studies: four of our variants (p.Gly1858Glu, p.Thr1866Met, p.Arg1890Cys and p.Tyr1942Cys) located in the ZP domain were identified in probands with a U-shaped audiogram. However, the p.Cys650Arg variant identified in the cysteine-rich TIL1 domain is responsible for mild HL, which became clinically manifest at the age of 16 years. The audiometric curve has a shallow U-shape profile. Similarly, Yasukawa et al.^37^ identified the likely pathogenic p.Cys606Gly variant in the TIL1 domain in a proband with mid-frequency HL. Variant p.Lys2150Asnfs*9 is located in the last exon and causes replacement of the last six amino acids and extension of the glycosylphosphatidylinositol (GPI) anchorage of TECTA protein by two amino acids. It has been shown that GPI-dependent release of TECTA is required for elongation and bundled organization of collagen fibrils on the apical surface of inner supporting cells during formation of the TM^38^. Therefore, any change in the conformation or length of the GPI anchorage may impair this process and lead to a clinical phenotype. However, further study of these mechanisms is required. In our study, 10% of all probands with a U-shaped audiogram carried pathogenic variants in *TECTA*, and when we also take into account all the identified VUS variants, the theoretical contribution of TECTA to hearing impairment in our cohort may be as high as 47%. This is more than the published prevalence of TECTA in patients with U-shaped audiograms, for example in Japan, where 6% of patients with U-shaped audiograms could be attributed to *TECTA*^36^. The difference may be explained by high variability in the prevalence of causal deafness variants and genes among different ethnic populations as a well-known phenomenon in hereditary HL^39^.

The *OTOGL* gene encodes an otogelin-like protein, which has structural similarities to the epithelial-secreted mucin protein family^40^. Its mutations lead to non-syndromic prelingual HL, which is mild to moderate and stable, with autosomal recessive inheritance^41^. The 2,343-amino acid protein contains an N-terminal signal peptide, four paired von Willebrand factor (VWF) and cysteine-rich (C8) domains, four trypsin inhibitor-like (TIL) domains, and a C-terminal cystine knot motif^40^. We identified two heterozygous variants in proband D1315. The first is the known pathogenic nonsense variant p.Gln520* in vWFD2^42^. The second likely pathogenic variant, c.4032_4054+30del, has not yet been published. It is located at the interface of the 34th exon and 34th intron and, according to HSF and MaxEnt, it can cause an alteration of splicing due to activation of several cryptic acceptor and donor sites. However, we cannot rule out exon skipping, which may result in a more significant shortening of the protein sequence located between domains vWFD3 and vWFD4. The exon skipping does not lead to a frameshift, and therefore, according to Abou Tayoun et al.^43^, only the PVS1_strong classification criterion can be applied. On the other hand, vWFD domains containing the multimerization consensus site CGLC are essential for the multimer assembly of proteins expressed in the tectorial membrane (like α-tectorin, otogelin or otogelin-like protein) to form a filament and higher order structures. Although little is known about these domains in OTOGL, mutations in the vWFDs of TECTA have been clearly connected with hearing impairment^37,44^. Moreover, the shape of the audiometric curve observed in our proband is almost identical to the audiogram obtained from the compound heterozygous patient carrying mutations p.Gln520* and p.Arg925* reported by Bonnet et al.^42^. The vestibular hypofunction observed in patients by Oonk et al.^45^ was not diagnosed in our proband.

### Collagens expressed in the cochlea

The collagen superfamily comprises 28 types (I–XXVIII) in vertebrates^46^. Collagens are fibrous structural proteins involved in the construction of skin, cartilage, bone, eye, and other tissues^47^. All collagen molecules are comprised of one, two, or three different types of α-chain subunits tightly wrapped into a triple helix. A single missense mutation of a glycine to another residue in the triple helix can result in almost 40 genetic diseases^46^. Collagens encoded by genes *COL2A1*, *COL4A3*, *COL4A4*, *COL4A5*, *COL4A6*, *COL9A1*, *COL9A3*, *COL11A1* and *COL11A2* are essential for proper auditory function. Mutations in these genes are linked to non-syndromic or syndromic HL (Marshall syndrome, fibrochondrogenesis, Alport syndrome, Stickler syndrome)^19,48^. In this study, we present a novel variant in the *COL4A5* gene. Variants in this gene are associated with X-linked Alport syndrome^49^. In 80% of all cases, Alport syndrome is caused by mutations in the *COL4A5* gene^50^. The disorder shows considerable heterogeneity in the age of onset of end-stage renal disease and the occurrence of deafness among particular families^51^. Most patients have mid-frequency HL, although high frequency and flat SNHL curves may be present, as well. SNHL in males may first manifest around the age of 10 years and usually gets progressively worse^49,52^. In family FAM618 we identified the variant p.Gly472Glu, which interrupts the Gly-Val-Lys triplet in the α-chain and results in disruption of the triple helix’s function, changing the scaffolding and flexibility of collagen IV^46^. This results in an Alport syndrome phenotype in both siblings (D815, D817).

### Genes expressed in hair cells

Myosins play an important role in the cellular organization of the cochlea. In general, myosins are motor proteins, and based on their variable C-terminal binding domains, they are included in conventional myosins (class II) and unconventional myosins (classes I and III to XV)^53^. Unconventional myosins IA, IIIA, VI, VIIA and XVA occur in the cochlea. *MYO7A* and *MYO15A*, encoding unconventional myosins, are expressed in the outer and inner hair cells of the Organ of Corti^54,55^.

*MYO15A* is known to be the third-most mutated gene causing severe to profound ARNSHL, after *GJB2* and *SLC26A4* mutations. Mutations in this gene cause both pre- and post-lingual forms of progressive HL^41,56^. Our patient D1321 clinically manifests profound non-syndromic HL. Interestingly, she passed the neonatal hearing screening, and her HL became apparent at the age of 1.5 years. We identified two variants, c.5693G>A and c.8050T>C, the latter of which was previously described in trans with other pathogenic variants^57,58^, whereas c.5693G>A variant was submitted only to the ClinVar archive and to the Deafness Variation Database (https://deafnessvariationdatabase.org/)^59^. It is located in the myosin motor domain of the *MYO15A* protein. This domain consists of ATP- and actin-binding sites, which can generate force and move actin filaments; therefore, motor domain dysfunction results in shorter stereocilia with an ectopic staircase structure of stereocilia associated with a severe deafness phenotype^60^.

The MYO7A tail contains a pair of myosintail homology 4-protein 4.1, ezrin, radixin, moesin (MyTH4-FERM) tandems separated by an SH3 domain. Mutations in the head and tail regions of MYO7A lead to defects in mechanotransduction and result in Usher syndrome 1B (USH1B), an autosomal recessive disorder with sensory impairment^61^. In addition to syndromic conditions, mutations in *MYO7A* can cause dominant (DFNA11)^62^ and recessive (DFNB2)^63^ non-syndromic HL. The p.Arg1338His variant detected in FAM411 is located in the FERM 1 domain of the tail of the myosin VIIA protein. The nonsense variants p.Arg1338AlafsTer61, p.Gln1336Ter or p.Glu1337SerfsTer62, linked to Usher syndrome, were identified close to our identified p.Arg1338His variant. VUS missense variants p.Arg1338Ser or p.Arg1338Cys with unclear clinical phenotype were also identified (https://deafnessvariationdatabase.org/). Our proband had HL onset at the age of 22 years with mild progression of hearing impairment and a U-shape audiogram. Moreover, no retinal abnormalities were recorded, indicating the dominant non-syndromic HL.

Transmembrane channel-like protein 1 is a protein encoded by the *TMC1* gene. The predicted structure of TMC1 shows a dimeric channel with 10 transmembrane domains (S1–S10) and intracellular amino- and carboxyl-termini in each monomer. Transmembrane helices S4–S7 form a groove that lines the pore of sensory transduction channels^64^. The transmembrane channels are proteins necessary for hair cell mechanotransduction^65^. Dominant mutations in *TMC1* cause the late-onset progressive HL phenotype, whereas ARSNHL cases are linked to congenital severe-to-profound HL^66,67^. We identified a missense variant, c.1949C>T, which is predicted to substitute a conserved proline at position 650 for leucine, which may influence the correct folding or assembly of TMC1 into multimers. It is located in the helical S9 domain. Two pathogenic autosomal recessive variants were previously identified in close proximity to our variant: p.Met654Val^68^ and p.Ser647Pro^69^. However, according to the phenotype observed in FAM416, we cannot rule out the dominant character of inheritance of the p.Pro650Leu variant or a yet undetected causal variant in a different gene. One young family member, D1337, showed still normal hearing, although she carried the same variant as her father, thus supporting the possibility of late-onset HL.

The *TMPRSS3* gene encodes a member of the serine protease family. Transmembrane Serine Protease 3 is linked to hair cell sterocilia mechanics and to actin network formation, which supports cell motility and integrity. Apart from hair cells, the *TMPRSS3* gene is also expressed in the inner and outer pillar cells and the Deiters cells, stria vascularis or ganglion spirale^70,71^. This gene was identified by its association with childhood onset of an autosomal recessive deafness. The hearing impairment in families with mutations in the *TMPRSS3* gene has a prelingual to postlingual onset. A common feature of patients with *TMPRSS3* mutations is progressive HL beginning in high frequencies and often leading to partial or complete deafness. However, Sasamori et al.^72^ reported a case of progressive mid- to low-frequency sensorineural HL associated with mutation of the *TMPRSS3* gene. We identified a likely pathogenic variant, p.Arg106Cys, which is located within exon 4 and causes the negatively charged residue arginine to change to a neutral hydrophobic cysteine at position 106. This change can result in the loss of hydrogen bonds and/or disturb the correct folding of TMPRSS3. It has been observed in the compound heterozygote state with p.(Phe13Serfs∗12) and p.Ala306Thr, where it cosegregated with prelingual profound hearing impairment or postlingual milder hearing impairment, respectively^70^. The homozygous form observed in proband D298 has not previously been observed. Although Gao et al.^70^ predicted a very subtle or no effect on auditory function of homozygous p.Arg106Cys, we have shown that it is associated with moderate to severe perilingual progressive HL with a typical U-shape audiogram.

The *DIAPH1* gene encodes human protein DIA1, a formin that elongates unbranched actin^73,74^. The correct polymerization of actin is critical for the formation and elongation of stereocilia on the apical surface of cochlear hair cells^75^. Expression of *DIAPH1* in the Organ of Corti has been proven in the inner pillar hair cells as well as at the base of the outer hair cells and in the outer pillar cells^76^. DIA1 consists of the GTPase-binding domain (GBD), a partially overlapping N-terminal diaphanous inhibitory domain (DID), formin homology (FH) domains FH1 and FH2, and the C-terminal DAD. It is held inactive in the resting state through an autoinhibitory intramolecular interaction between the DID and the DAD, which is regulated by Rho family GTPases^74^. In this study, we identified the VUS variant p.Gly1197Asp which, according to the Human Splicing Finder, has no significant impact on splicing signals. It is located in close proximity to the MDxLLxxL recognition site (1199-1206aa), which presents the central consensus motif of the DAD domain and is essential for binding to the DID domain. We hypothesize that our missense c.3590G>A variant results in a change from a small, uncharged amino acid glycine to a larger, charged residue aspartate. This may have a profound effect on the conformation of the amphipathic helix of the DAD domain and subsequently impair interaction with the acidic groove of the DID. Disruption of the autoinhibitory intramolecular DID-DAD interaction and consequent activation of DIA1 may explain the DFNA1 pathology^74^. *DIAPH1* mutations have been associated with initially mild, low-frequency SNHL, characterized by progressive deafness starting in childhood^73^. In contrast, some described variants showed hearing impairment beginning in the high-frequency range and progressing to deafness involving all frequencies^74^. Audiograms of our proband and family members showed a typical U-shape. Autosomal dominant deafness caused by mutations in the *DIAPH1* gene can be associated with thrombocytopenia^76,77^. However, in the affected family members D1248, D1249 and D1250 no thrombocytopenia was confirmed suggesting non-syndromic HL. The only pathological finding identified by differential blood count analysis was neutropenia in the family member D1250.

The *TSPEAR* gene encodes the thrombospondin-type laminin G domain and EAR repeats protein (TSPEAR), which was detected at the basal region of the stereocilia of hair cells. Mutations in *TSPEAR* cause non-syndromic SNHL with prelingual onset^78^. We identified the nonsense variant p.Glu624* in exone 12 resulting in a loss of the EAR7 repeat. Seven epilepsy-associated repeats (EARs) of *TSPEAR* are predicted to form a beta-propeller structure, function as part of a ligand-binding domain and mediate protein–protein interactions^79^. Delmaghani et al.^78^ identified in 3 affected brothers from a consanguineous Iranian family segregating autosomal recessive nonsyndromic sensorineural deafness a homozygous frame-shift mutation (c.1726G>T+c.1728delC) that was predicted to result in termination of translation in the EAR6 repeat, connected with defective secretion of the mutant protein and congenital profound sensorineural deafness. As both affected siblings in our family FAM174 segregated a similar HL phenotype, but only one of them carried the homozygous variant in *TSPEAR*, we may assume that in addition to the identified p.Glu624* variant in the *TSPEAR* gene a second, yet unknown gene may contribute to HL in this family.

In general, autosomal recessive variants are mainly responsible for prelingual forms of severe to profound hearing impairment, whereas autosomal dominant variants cause HL that usually appears later and is often mild or moderate. Exceptions comprising postlingual HL include recessive variants in the *TMPRSS3* gene; prelingual include dominant variants in the *TECTA* gene^32^.

The precise data on the prevalence of pathogenic variants in the genes described above in the general and hearing impaired populations in Slovakia are not yet available. Most of the variants were found in single families, although pathogenic variants in *TMPRSS3* were also identified in two probands with typical ski-slope audiograms (unpubl. data).

### Conclusions

This study confirms the genetic background in at least 20% of patients with mid-frequency SNHL. Two of the identified genes (*TMPRSS3* and *MYO15A*) are reported in association with this audiogram configuration for the first time. Moreover, we attempted to narrow the spectrum of candidate genes in this specific and rare SNHL form to 3 distinct groups based on the site of their expression and their function in the cochlea. In our opinion, Sanger sequencing of *TECTA* or WES are currently two cost-effective options for DNA analysis of mid-frequency SNHL.

## Methods

### Patients recruitment

Patients with mid-frequency HL were selected from among 851 families (1074 hearing impaired individuals) with bilateral SNHL with onset before the age of 60 years whose DNA samples and clinical data were collected throughout the whole Slovak Republic over the last decade. The subjects were referred from major ENT departments in Bratislava or recruited at boarding schools for hearing-disabled children and in selected isolated Roma communities. Peripheral blood and/or buccal swabs were taken for DNA isolation. The *GJB2* gene was analyzed in all probands first to exclude the most frequent cause of hereditary HL. Only non-*GJB2* associated cases (71% of all probands) were considered for further analyses. All individuals or their legal representatives (in subjects under 18 years of age) signed informed consent to genetic testing and the study was approved by the Ethics Committee of University Hospital in Bratislava.

Mediocochlear HL was defined by the highest pure tone thresholds in mid-frequencies (1–4 kHz). In typical cases, the difference between the worst mid-frequency threshold and the high- and low-frequency thresholds was at least 20 dB. However, individual variability of the audiometric curve shapes was relatively common among the patients and sometimes also between the two ears of the same individual. Borderline cases with shallow curves were included only if the mid-frequency HL was confirmed to be reproducible during the audiometric follow-up. Preschool children and subjects in whom reliable audiometric data were not available were excluded from the study. Based on the criteria mentioned above, we selected 30 probands (27 of Caucasian and 3 of Roma ethnicity) with mid-frequency HL of yet unknown aetiology.

### Whole exome sequencing

Probands meeting the inclusion criteria were selected for WES analysis. Genomic DNA was extracted from peripheral blood using standard procedures. WES was performed by service providers: BGI, Hong Kong, and Theragen, Republic of Korea. A DNA library was prepared using the BGI 59M Human Exome kit (probands D527, D753, D815, D821 and D822) and the SureSelect XT V6 kit (all remaining probands), respectively. Sequencing data were processed by the provider’s standard bioinformatics pipeline, which encompassed base calling and alignment of generated reads to the GRCh38 reference genome without the unplaced or alternate loci. Variant calling was routinely performed in-house using The Genetic Analysis Tool Kit (GATK) HaplotypeCaller, combined with the GATK UnifiedGenotyper in the case of BGI samples^80^.

Aligned reads and called variants were obtained in standard bioinformatics formats and subjected to the following bioinformatics pipeline. Variants were decomposed and normalized using vt^81^. Variant effect predictor^82^ was used to annotate the variants with respect to their potential effects on genes and transcripts and to add scores from in silico prediction algorithms PolyPhen and SIFT^83^. Finally, the Gemini framework^84^ was used for submitting variants into a newly created SQLite database and for annotating variants with additional data from genome annotation databases, e.g. the Single Nucleotide Polymorphism Database (dbSNP)^85^, Encyclopedia of DNA Elements (ENCODE) (The ENCODE Project Consortium), ClinVar^86^, 1000 Genomes (The 1000 Genomes Project Consortium), the Exome Sequencing Project (ESP) (http://evs.gs.washington.edu/EVS/), Exome Aggregation Consortium (ExAC) and the Genome Aggregation Database gnomAD^87^. Afterwards, variant prioritization and filtering were carried out by removing those variants with an MAF ≥ 0.01 (based on maximal MAF in all used population databases), and the resulting variant set was first narrowed down by removing variants not lying inside the regions of 226 genes with known association with SNHL. The set of evaluated SNHL genes was based mainly on the webpage https://hereditaryhearingloss.org/. In the case that no candidate variants were found among the virtual panel of 226 genes, the whole exome was evaluated. Candidate variants were clinically classified according to the American College of Medical Genetics and Genomics (ACMG) guidelines^88^ adapted by Oza et al.^89^, with modification for the PVS1 criterion according to Abou Tayoun et al.^43^. The VUS variants were selected as candidates only if they belonged to previously reported deafness genes. The limited number of available family members needed for cosegregation analysis makes it difficult to classify new gene variants among novel genes. Moreover, the further classification of VUS variant in unknown genes would be inconclusive without functional studies.

### Sanger sequencing

Sanger sequencing was performed to verify variants identified by WES and to determine co-segregation of the candidate variants with HL in all participating family members. The primers were designed using the program Primer Blast^90^. The resulting PCR products were sequenced using the Big Dye Terminator v3.1 kit and analyzed with the ABI3500 Genetic Analyzer. Sequence chromatograms were analyzed by Sequence Scanner Software 2.0 (available from http://www.thermofisher.com/).

All methods were performed in accordance with the relevant guidelines and regulations. Copy Number Variants (CNV) were not investigated. Some of the unsolved families could be due to CNVs.
